# Addressing gendered varietal and trait preferences in West African maize

**DOI:** 10.1016/j.wdp.2020.100268

**Published:** 2020-12

**Authors:** Amare Tegbaru, Abebe Menkir, Mohamed Nasser Baco, Latifou Idrisou, Dioukou Sissoko, Ayinde O. Eyitayo, Tsedeke Abate, Abdoulaye Tahirou

**Affiliations:** a International Institute of Tropical Agriculture (IITA), Sweden; b Faculté d'Agronomie (FA), Université de Parakou, Benin; c Centre régional de recherche agronomique de Sotuba, Mali; d Faculty of Agricultural Economics, Ilorin University, Nigeria; e Abate Tsedeke, Former Project Manager of CIMMYT-STMA, Addis Abeba, Ethiopia

**Keywords:** Maize, Breeding, Gender, Varieties, Traits, Partnerships

## Abstract

Maize is the most important cereal in Sub Saharan Africa; however, yields are significantly lower than those possible with improvements in cultivars and management. Maize breeding programs need to produce material with improved resistance to increasing environmental stresses and incorporate the man and women farmer preferred traits that relate to yield, postharvest, nutritional, and processing qualities. This research uses gender-disaggregated data recorded during participatory on-farm maize trials by the Stress Tolerant Maize Program conducted in agroecological zones of Benin, Nigeria and Mali) to identify men and women farmer’s expressed varietal and trait preferences in order to evaluate plant breeding strategy. A multi-stage varietal and trait identification process was used to identify gender shared and distinctive varietal trait preferences for product development and dissemination. The data indicates that progress has been made by the Project in considering the range of traits valued by both men and women farmers and indicates those that should be considered for gender-focused product pipeline development in the future. The study concludes by underlining the need for adjustment in breeding to improve partnerships with food scientists, postharvest specialists and private seed sector on the packaging and delivery of technologies to farmers and other value chain actors.

## Highlights

• No evidence to determine whether maize breeding programs has been favouring or disfavouring men or women farmers.
• No clear distinction between most of the drought tolerant maize varieties and hybrids preferred by men and women farmers.
• The inherent properties of the hybrids and open-pollinated maize varieties were the drivers of preferences of men or women farmers.
• Better breeding strategies that permit the development of gender-response products with greater potential impact on livelihoods.
• Need for better breeding strategies that permit the development of gender-response products with greater potential impact on improved food and nutritional security.
• Market opportunities exist for private seed companies to ensure th constant supply of seed for women and men user groups and sustain the seed value chain.

## 1 Introduction

World population is rapidly increasing, and global food production must increase to address that population’s needs (UN, 2017). At the same time, the world is facing changes in climate that challenge both the productivity and sustainability of agricultural activities (Haussmann, 2012). The development of new technological innovations and their adoption by producers is vital to address this challenge. (Wheeler and von Braun, 2016, Aarts et al., 2014, Sewell et al., 2017). This paper examines the development of drought/ tolerant maize varieties in West Africa. The focus is on the identification of gender preferred traits that will improve the packaging and delivery of appropriate maize technologies to farmers and other value chain actors. Maize is the most important cereal and most widely cultivated staple crop in Sub Saharan Africa. It is a strategic and multiple purpose crop for the more than 208 million people who depend on it for food security and economic well-being in West Africa (see Abate et al 2017). It occupies the largest land area of all staples in the region and annual maize grain production is estimated at nearly 72 million metric tons (MT). However, average yields of maize in this area are considerably lower than those obtainable with improved cultivars and management and low cultivar turnover is a significant contributing factor (Abate et al., 2017) (Table 1).

**Table 1:** Combined respondents.

	Male	Female	Total
Nigeria	101	100	201
Benin	20	20	40
Mali	65	65	130
Total	186	185	371

Decisions by men or women farmers to replace an existing maize variety with a new one depend upon their perception and experience of the benefits the new variety may provide (see Everson and Gollin, 2003, Laborte et al., 2015). Knowledge of trait characteristics preferred by men and women farmers, as well as other actors in the value chain, enables definition of new variety product profiles with a greater potential for adoption. The analysis informs the attractiveness of the market in terms of measurable customers, profitability, response to differentiated products and stable returns to justify investment (see Ahlman et al., 2014, Laborte et al., 2015, Slagboom et al., 2016; Christinck, 2017).

Gender has significant impacts on farming/ agricultural activities. Men and women have unequal access to and control over key productive resources upon which agriculture depends. Gender differences also affect how crops are utilized in postharvest and food processing and marketing and how these are valued by different consumer groups (Weltzien, Rattunde, Christinick & Ashby, 2020: 246–247). Studies have shown that farmers’ preferences for traits are diverse, depending on factors such as farm characteristics, production systems and farmers’ production objectives (Ashby et al., 1994, Meinzen-Dick et al., 2007, Slagboom et al., 2016, Laborte et al., 2015). Women and men can select and grow the same crop varieties under similar or different circumstances for various reasons (Weltzien et al., 2020). They differ in trait preferences when they face different constraints, different roles and responsibilities in production and consumption systems, and different crop production goals (Christinck et al., 2017). Women’s varietal trait preferences appear more frequently related to food security traits such as early maturity, postharvest processing and food preparation including storability, grain colour and texture (Weltzien et al., 2020). Women are also attracted to certain critical characteristics related to family food security such as earliness, multiple harvests, production potential during full growing seasons as well as productivity under sub-optimal soil fertility (Christinck et al., 2017). For some time breeding programs have recognized the need to consider gender differences in trait preferences and incorporate them in programs (Grando, 2017). However, there is still a lack of innovative and applicable techniques that that can be readily employed to effectively identify the gender dimension in their programs and their implications for improving breeding schemes (see Orr et al., 2018).

This paper outlines an approach to examine the sex-disaggregated data recorded in multi-year participatory on-farm trials of maize in three selected West African countries (Benin, Nigeria and Mali) by the Tolerant Maize Project (STMA) funded by the Bill and Melinda Gate Foundation. The research employs farmers’ responses to varietal and trait or other characteristic preference selections and uses the results of the analyses to identify specific gender preferred characteristics that relate to postharvest, nutritional and processing qualities with implications for future breeding of maize varieties appropriate for both male and female farmers.

The term ‘traits’ as used among plant breeders generally refers to identifiable varietal aspects that can be measured and directly taken up by breeders themselves. Although some characteristics coincide with these kinds of 'traits' many others do not (e.g. good for food) and therefore ‘traits’ is used while referring to varietal selections and 'characteristics' employed when referring to preferences related to marketing, taste, colour, postharvest food processing, etc.

### 1.1 Study context

Under the STMA, project stress resilient elite open-pollinated maize varieties (OPVs) and hybrids were included in regional trials and shared with the national partners for extensive testing in their countries. The national partners select the most promising stress resilient OPV and hybrids adapted to their conditions for further evaluation in participatory on-farm trials. It was recognized that these trials could be used as platforms to identify preferred traits of women and men small holder farmers that can be targeted by breeders to develop products attractive to the two groups. Consequently, participatory on farm trials involving the selected stress resilient OPVs/hybrids were planted alongside with farmers’ preferred maize variety and used as platforms where both mean and women farmers closely assessed the varieties and provided their preferred varieties and traits to the researchers.

The study is undertaken in the context of the Drought Tolerant Maize for Africa (DTMA) and Drought Tolerant Maize for Africa (STMA) projects in West Africa that focus on addressing multiple constraints affecting maize production including the adverse effects of drought, climate change and low cultivar replacement (Abate et al., 2017, Ayedun, 2017, Shiferaw et al., 2011, Fisher et al., 2015). These projects involved the development, release, multiplication, deployment and monitoring of drought tolerant maize varieties in each country as well as the constraints impacting their adoption by farmers.

A comprehensive strategy to integrate gender into varietal selection, identification of trait preferences, product development, and deployment was only developed and implemented in the final phase of the DTMA project in 2012. Building upon the learnings of the DTMA project, the STMA project encouraged a systematic documentation of men’s and women’s varietal preferences with special emphasis on the sets of characteristics that met consumption needs and addressed constraints of women farmers; access to quality seed, labour and the drudgery associated with production with data collected by project partners in Benin, Mali and Nigeria. Gender-focused approaches on drought and drought resilient varietal development have since been incorporated into the first phase of the Tolerant Maize for Africa (STMA) project expanding upon the legacy of DTMA’s significant contributions.

Since the STMA project launch in 2016 greater emphasis has been placed on increasing the impact of drought tolerant maize varieties through enhanced public–private partnerships and allocating resources to address gender-focused and livelihood-related concerns such as food security, nutrition, drought and postharvest issues. The project had four major outcomes (or objectives) (STMA, 2016). These were:

Innovative breeding tools and techniques to increase the rate of genetic gain in the maize breeding pipeline;
Increased commercialization of improved multiple-drought-tolerant maize varieties with gender-preferred traits by the sub-Saharan African seed sector;
Delivery and out-scaling of released and new drought tolerant maize varieties through strong public–private partnerships; and
Project management, monitoring and evaluation, and communication.

Investment to narrow gender inequity gaps featured in a large proportion of program efforts in the third Outcome (obejective) and took a range of different forms: a commitment to supporting women-owned seed companies; employing gender-responsive approaches to promote and stimulate demand for newly released drought tolerant maize varieties to speed up variety turnover; developing and implementing a product dissemination plan, particularly targeting women and disadvantaged groups; strengthening the capacity of seed companies to deliver improved seeds particularly to women; supporting effective participation of women in seed value chains, and building knowledge on gender and maize-legume seed value chains (Rubin, 2015).

The project also carried out gender capacity assessment for partners and provided technical support and training on gender integration in seed business development. A pilot gender certification standard for the seed sector was also developed.

### 1.2 Dataset characteristics

The On-Farm Trial dataset used in this study was obtained from the principal maize producing agro-ecological zones in Nigeria, Benin and Mali. These data sets were developed to identify differences as well as similarities in varietal and trait or other consumer based characteristic preferences that can guide gender-responsive approaches to enhance the demand for drought and drought tolerant maize varieties by both men and women famers.

The data for Nigeria and Mali cover the period of 2013, 2014, and 2015 while the Benin data from the Northern Guinea Savanna is for 2014, 2015 and 2017. In each country standardized and harmonized semi-structured individual interviews and focus group discussions were employed in each of the study countries to collect on-farm trial data such as gender differentiated perceptions of the varieties, preferences of traits, and other consumer-based characteristics related to postharvest uses and benefits.

### 1.3 Nigeria

Nigeria alone produces about 43% of maize grown in West Africa and women constitute 49.5% of the labour force in maize as well as legume and cassava based farming systems. (NBS, 2016). The crop is grown in virtually all the agroecological zones of the country. The Northern and Southern Guinea Savannah, where the on-farm trail data was collected are the largest maize growing areas of Nigeria. The data was collected from poor and resource limited men and women members of the farming households in the more marginal rain-fed agricultural areas of the Northern and Southern Guinea Savannah targeted by the Drought Tolerant (DT) Maize for Africa (DTMA) and Drought Tolerant Maize for Africa project (STMA). It covers the period of 2013, 2014 and 2015.

In Nigeria alone more than 40 DT open pollinated varieties (OPVs) of maize have been tested in farmers’ fields, either as a combination of Mother and Baby trials or as on-farm trials, in which two DT OPVs are tested in farmers’ fields with the farmer’s variety as the check. This was undertaken in three states (Kaduna, Borno and Taraba) in the Northern Guinea Savannah (NGS) and three states (Kwara, Niger and Oyo) in the Southern Gunnea Savannah (SGS). The average annual rainfall within the study areas is 1500 mm (Ayanlade, 2009). The Mother-Baby approach tests a number of different technologies (in this case the DT maize varieties) in a central location (the Mother trial) while the Baby trial tests a subset of the technology, which in this case consisted of two of the DT varieties along with a farmer’s variety (control) in farmers’ fields, scattered around the central Mother trial location.

### 1.4 Benin

In Benin, the study was conducted in the northern part of the country. It is characterized by a diversity of maize-based production systems with generally low productivity. This is mainly due to poor physical conditions such as a decline in soil fertility coupled with less than optimal fertilizer use and a low level of adoption of improved varieties and other productivity enhancing technologies. Maize farmers in northern Benin rely mainly on recycled traditional seeds with limited potential which contributes to the low productivity of traditional farming systems (Vernooy, 2003). The women population represents 51% to 53% (INSAE 2017 of the agricultural labour force. Despite recent subsidized programs in some countries, a lack of access to good quality fertilizers has also hindered farmers abilities to achieve the full potential of their maize farming operations.

Within the study zone, average rainfall is low (950 mm) (Ibouraïma & Afouda, 2010) and changes dramatically from month to month, significantly affecting agricultural production. The Department of Alibori was chosen to conduct this study. According to Agbossou et al. (2010), the department has the greatest potential vulnerability to climate change in Benin. The selected sites were chosen because of their low level of productivity of maize and also because maize is viewed as the most sensitive food crop to the effects of climate change (Agbossou et al., 2010). In each selected village, five varieties were tested. Four of the tested varieties were drought tolerant. The fifth, a non-drought tolerant variety commonly planted in the village, served as a control.

Producers who hosted the trials were selected in collaboration with technicians of the National Agricultural Research Institute of Benin (INRAB). At each site, 10 women and 10 men were selected to participate in the trial.

### 1.5 Mali

The data from Mali were obtained from the Sudan Savannah, the largest maize growing agro-ecological zone in the country and covers the period of 2015, 2016 and 2017. Mali has four main agro-ecological zones. The Sudan Savannah with 600–1100 mm rainfall (Akinseye et al., 2016) accounts for about 70% of the total maize area. Major maize growing administrative regions include Sikasso (59%), Koulikoro (17%), Kayes (16%), and Ségou (7%). Women constitute 50.4% of the labour force (Enquête modulaire, INSTAT, 2015). In terms of land use, maize and cotton are the main crops, each occupying about 30% of the land under cultivation. They are followed by cereal associations (sorghum, maize, millet, etc.), legumes (peanuts, cowpeas, soybeans, Bambara groundnut, etc.), sorghum, the maize-sorghum complex, peanuts, etc. Intercropping of cereal and legumes remains a common practice. However, land coverage of maize is expanding and is presently the dominant crop followed by cotton.

In summary, in the Northern and Southern Guinea of Nigeria and Benin, and the Sudan Savannah of Mali maize production has increased in recent years. The general upward trend in maize production could be explained by the increasing use of inputs (fertilizers, herbicides and improved varieties).

## 2 Methodology

Under the STMA, project stress resilient elite open-pollinated maize varieties (OPVs) and hybrids were included in regional trials and shared with the national partners for extensive testing in their countries. The national partners select the most promising stress resilient OPV and hybrids adapted to their conditions for further evaluation in participatory on-farm trials. It was recognized that these trials could be used as platforms to identify preferred traits of women and men small holder farmers that can be targeted by breeders to develop products attractive to the two groups. Consequently, participatory on farm trials involving the selected stress resilient OPVs/hybrids were planted alongside with farmers’ preferred maize variety and used as platforms where both men and women farmers closely assessed the varieties and provided their preferred varieties and traits to the researchers.

**Fig. 1:**
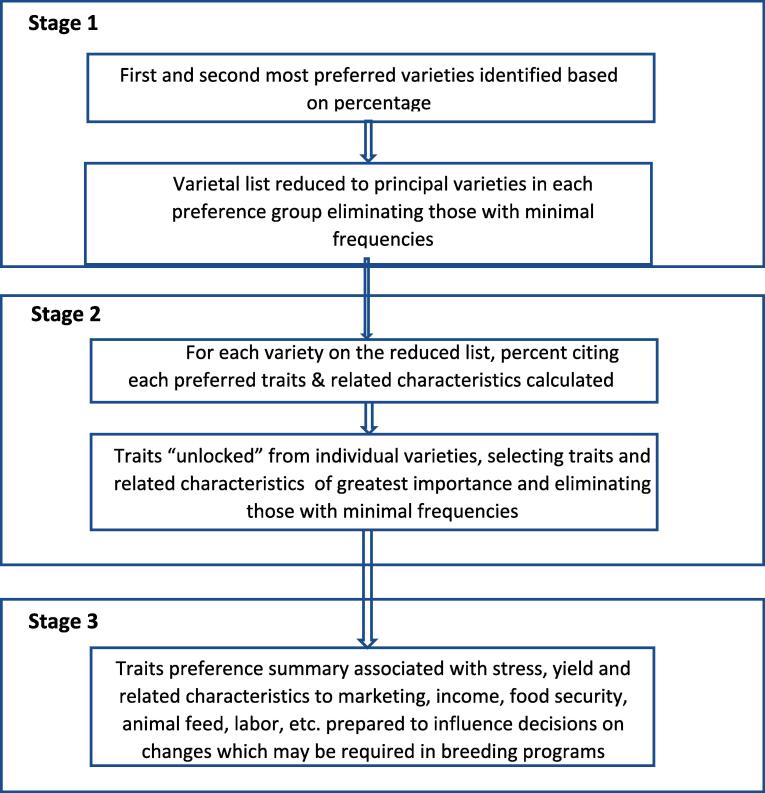
Multi-stage varietal and trait identification procedure.

The present study used a three-stage process (Fig. 1). In Stage I, farmers identified their first and second most preferred varieties. In Stage II, the first, second and third most important preferred traits or other preferred characteristics of participating men and women farmers were selected. A summary of key preferred traits and postharvest and food processing characteristics that should be considered for breeding for the gender-focused product pipeline development in the future are presented in Stage III.

### 2.1 Farmer preferred varietal selection procedure

During the participatory on-farm trial visits, respondents were asked to indicate their first and second preferred maize varieties. In each country and separately for men and women the varieties selected were listed in descending order based upon the proportion selected. Varieties selected by fewer than 20% of both men and women in Nigeria, 10% of men and 20% of women in Benin and 16% of men and 22% of women in Mali were then eliminated from the lists to retain just the predominantly selected varieties.

### 2.2 Key preferred characteristic identification of the selected varieties

For each selected variety, respondents were asked to indicate the visible traits which they considered important to their selection. The percentage of men and women respondents preferring each identified trait was then calculated and characteristics with less than 15% for both women and men in Nigeria, 14% for men and 12% for women in Benin and 11% for both women and men in Mali were eliminated.

### 2.3 Results

In this section, the first and second ranked maize varieties and hybrids identified by male and female farmers in the agroecological zones of each country are identified and the respondent’s preferred characteristics described. The Distinct preferences reported in each table are those varieties identified only by men or by women in that region.

The results for both the North and South Guinea agroecological zones of Nigeria are reported in Table 2.

**Table 2:** Nigeria – Northern and Southern Guinea Savannah.

First ranked varieties							Second ranked varieties						
Women & Men	Men	Women	Women’s distinct preference (b)		Men’s distinct preferences (c)		Women & Men (d)	Men	Women	Women’s distinct preferences (e)		Men’s distinct preferences (f)	
Northern Guineas Savannah													
12C7685*	33	5	STR-SYN-Y1**	11	M1124-8*	35	EEWH-2*	18	4	Ayba-Kubwa (local check)**	8	11C86*	27
EHW-1*	27	5	EWH3*	7	TZL COMP1 SynW-1**	23	9002–13*	10	6	M1327-20*	5	Z.DIPLO.BC4C3-W-DT C1**	12
M1328-9*	9	6					12C7685*	7	4				
EWH-27*	7	3					M1328-10*	8	3				
M1327-47*	7	3					10C8447*						

Southern Guinea Savannah													
TZE DTSTR C4**	14	12	IWDC3 SYN × White DT STR SYN-DT C1**	7	None		2009 DTE Y STR**	11	11	IWDC3 SYN × White DT STR SYN-DT C1**	5	DY STR SYM WD C3**	5
TZEEI 81X TZZ EE 95*	8	12	TZ COMP ZDPSYN**	4			99TZEE Y STR**	5	4				
TZEEI 95 X TZZEE 58*	8	8					M102613*	3	6				
DT STR SYM WD C3**	10	10					2013 TZEE WDT STR**	2	6				
EVDT99**	7	9					DT STR SYM WD C3**	16	9				

*Refers to Hybrid = freshly purchased hybrid seed. ** **Refers to Open Pollinated Varieties (OPV)** = seed that has not been recycled for more than three seasons.

In the Northern Guinea Savannah zone, five drought tolerant maize hybrids were selected as first and second preferred varieties by women and men respondents. At the same time, few drought tolerant open pollinated (OPV) maize varieties and hybrids were uniquely preferred and ranked first and second by either men or women farmers. Most of the products ranked first and second by men and women farmers in Northern Guinea Savannah were drought tolerant maize hybrids. The underlying reasons behind these preferences are that the open pollinated and hybrids are both drought tolerant. However, seed of open pollinated maize can be saved at harvest and be re-planted by small scale producers whose resource endowment limits access to hybrids. It can also be multiplied and produced by the national seed production system and distributed to farmers in times of seed scarcity and food shortage. OPV’s can respond to inputs but not as effectively as hybrid varieties. The hybrid has high yield potential and drought tolerance. Even though both these varieties are endowed with consumption and marketable characteristics, men value hybrids more for their marketability. Hybrids are also preferred by private sector partners who, in the long run, are the drivers of constant and sustainable seed supply systems.

In the Southern Guinea Savannah of Nigeria (Table 2), two drought tolerant maize hybrids and three open-pollinated varieties were ranked first by both women and men farmers (see Column a), and three drought tolerant open-pollinated maize varieties and a hybrid ranked second by men and women farmers (see Column d).

### 2.4 Benin

Men and women respondents in the North Guinea Savannah of Benin (Table 3) ranked four open-pollinated maize varieties as their first preferred products (column a) and an open-pollinated variety as the second preferred product (see Column d). Women specifically ranked a drought tolerant maize hybrid as their first preferred product (see column b) and two drought tolerant open-pollinated maize varieties as their second (see column e). In contrast men did not specifically rank any of the drought tolerant maize varieties or hybrids in either their first or second preference list.

**Table 3:** Benin – Northern Guinea Savannah.

First ranked varieties						Second ranked varieties						
Women & Men (col a)	Men	Women	Women’s distinct preferences (col b)		men’s distinct preferences(col c)	Women & Men (col d)	Men	Women	women’s distinct preferences (col e)		Men’s distinct preferences (Col f)	
DSTR-WsynF2**	16	14	EVDT97STR**	6	None	2000Syn EEW**	2	6	Tzecomp3DTC1F2**	4	FAABA/QPM**	6
TZEE-Wpop-STR-QPMCO**	16	8							IWDC3syn/DTsyn-1-W**	4	EVDT-Y2008STR**	2
EVDT-Y2000STR**	18	6									2008SynEEWDTSTR**	2

### 2.5 Mali

In Mali (Table 4), five drought tolerant open-pollinated maize varieties and two hybrids were ranked first by both men and women farmers (see Column a) and three drought tolerant open-pollinated maize varieties and three hybrids were ranked second by both men and women farmers (see Column d). Men and women farmers did not identify any distinct and specifically preferred first and second ranked drought tolerant maize varieties or hybrids in Mali.

**Table 4:** Mali – Sudan Savannah.

First ranked varieties					Second ranked varieties		
Women & Men(col a)	Men	Women	Women’s distinct preferences(col b)	Men’s distinct preferences(col c)	Women & Men(col d)	Women’s distinct preferences (col e)	Men’s distinct preferences (Col f)
Témoin	3	3	None	None		–	–
V4:NAFAMA**	16	15	–	–		–	–
PVA Syn 11F2**	10	8	–	–		–	–
PVA Syn13**	6	4	–	–		–	–

### 2.6 Trait and related characteristics preferences of men and women farmers

A summary of farmer preferred traits and consumption related characteristics associated with the varieties selected in Nigeria, Benin and Mali are presented in Table 5, Table 6, Table 7. The selections reflect both shared and differing factors influencing specific production contexts; bio-physical environment, socio-economic circumstances, ethnic and cultural background and the prevailing farming systems (see Table 8).

**Table 5:** Trait preferences summary – Nigeria.

Traits & chacterstics	Overall Trait Rank	Number of Women (n)	Women %	Women’s rank	Men %	Men’s rank	Number of Men (n)
Big cob	1	25	25.23	1	32.13	1	32
Good appearance	2	19	18.89	1	6.74	3	7
Big stalk	2	16	16.34	2	13.60	2	14
Good for food	3	13	13.35	2	5.57	3	6
Colour	2	9	8.9	3	20.86	1	21
Good grain	8	7	7.29	3	0		
Marketable	4	4	4.3	4	7.87	2	8
Full cob	5	3	2.7	5	1.0	5	1
Drought tolerant	5	2	1.5	6	2.2	4	2
Early maturity	6	1	1.1	7	1.0	5	1
Multiple cobs		1	0.4		0		
Striga resistant	7		0		9.03	2	9
**Total**		**100**	**100**		**100**		**101**

**Table 6:** Trait preference summary – Benin.

Traits & characterstics	Overall Trait Rank	Number of Women (n)	Women %	Women’s rank	Number of Men (n)	Men %	Men’s rank
Yield	1	4	18.47	1	4	20.57	1
Striga resistant	1	3	15.98	1	4	21.66	1
Early maturity	2	2	12.44	2	3	14.47	2
Colour		2	12.80	2	0	0	
Drought tolerant	3	2	8.78	3	3	13.25	2
Big cob	3	1	6.75	3	3	13.94	2
Good appearance	7	1	6.91	3	0	0	
Plant height	4	1	5.15	3	2	7.78	3
Postharvest and shelf-life	8	1	3.66	5	0	0	
Organoleptic quality	8	1	3.66	5	0	0	
Well-filled grains	5	1	2.7	6	1	3.89	4
Pest and disease resistant	6	1	2.7	7	0	0	5
Food taste		0	0		0	0	
Robust plant		0	0		0	0	
Starch content	7	0	0		1	4.44	3
Smaller grain			0		0	0	
Weak density			0		0	0	
Good vegetation			0		0	0	
**Total**		**20**	**100**		**20**	**100**

**Table 7:** Trait preference summary - Mali.

Traits & characteristics	Overall Trait Rank	Number of Women (n)	Women %	Women’s rank	Number of Men (n)	Men %	Men’s rank
Drought tolerant	1	9	13.53	1	12	17.72	1
Cob full of grain	2	8	11.79	1	7	10.59	2
Big and long cob	3	7	11.34	1	3	5.05	3
Low cob placement	2	6	9.17	2	7	11.2	1
Resistance to lodging	2	6	9.17	2	7	11.2	1
Plant height	3	5	7.8	2	5	8.3	2
Yield	4	5	7.36	3	4	5.74	3
Early maturity	4	5	7.35	3	4	5.55	3
Big stalk	5	4	5.92	4	3	4.54	3
Double cob per plant	4	2	3.49	4	6	9.13	2
Grain colour	7	2	3.49	4	0	0	
Need less fertilizer	6	2	3.49	4	1	2.29	4
Yellow grain	6	2	3.49	4	1	2.29	4
Resistant to wind	8	2	2.6	5	2	2.29	
Earliness	7		0.00		1	2.29	4
Well covered	8		0		1	0.91	5
Middle cob placement	8		0		1	0.91	5
**Total**		**65**	**100**		**65**	**100**

**Table 8:** Regional overview of observed frequencies.

Chi-Square test									
Observed-freq	Observed Freq			Expected Freq			Chi-Sq Statstic		
Traits	Number of Women (n)	Number of Men (n)	Grand total	Number of Women (n)	Number of Men (n)	Grand total	Number of Women (n)	Number of Men (n)	Grand total
Big cob	25	32	57	28.36	28.64	57	0.40	0.39	0.791427827
Good appearance	19	7	26	12.94	13.06	26	2.84	2.82	5.658648134
Big stalk	16	14	30	14.93	15.07	30	0.08	0.08	0.153980198
Good for food	13	6	19	9.45	9.55	19	1.33	1.32	2.649134966
Colour	9	21	30	14.93	15.07	30	2.35	2.33	4.681455446
Good grain	7		7	3.48	3.52	7	3.55	3.52	7.07
Marketable	4	8	12	5.97	6.03	12	0.65	0.64	1.293861386
Full cob	3	1	4	1.99	2.01	4	0.51	0.51	1.020024752
Drought tolerant	2	2	4	1.99	2.01	4	0.00	0.00	9.90099E-05
Early maturity	1	1	2	1.00	1.00	2	0.00	0.00	4.9505E-05
Multiple cobs	1		1	0.50	0.50	1	0.51	0.50	1.01
Striga resistant		9	9	4.48	4.52	9	4.48	4.43	8.910891089
**Grand total**	**100**	**101**	**201**	**100.00**	**101.00**	**201**	**16.70**	**16.54**	**33.2395723**

P-value	
DF	10
# rows	10
# col	1
Critical value at 5% (0,05)	18.3070
P value	0.0099

In Nigeria (Table 2) the top three ranked characteristics include drought tolerance, grain yield and its related characteristics, maturity cycle, characteristics associated with consumption and animal feed as well as marketing. Income and food security characteristics dominated the Nigerian preference summary.

In Benin (Table 3), the top ranked characteristics were drought tolerance, yield and its related characteristics, maturity cycle, characteristics associated with consumption, animal feed and fuel. The Benin trait summary primarily addresses food security.

The most preferred characteristics in Mali (Table 4) include drought tolerance, ease of harvesting, yield and its related characteristics, maturity cycles, characteristics associated with animal feed and fuel. The selected characteristics highlight the importance of food security, use of labour at harvest time and the raising of domestic animals in producers’ communities.

#### 2.6.1 Summary of key characteristics for product pipeline development

The final stage of the analysis was summarizing the preferred characteristics associated with the selected varieties by men and women in the study countries (Table 5). The top three ranked characteristics are associated with drought tolerance, grain yield and its related characteristics, maturity cycle, and characteristics associated with marketing, income, food security, labour requirement use at the time of harvesting and animal feed.

Decision: so reject the overall ranking between men and women is the same at 1% significance level.

## 3 Summary of results

The on-farm trial data from Nigeria, Benin and Mali did not show a clear distinction between most of the drought tolerant maize varieties and hybrids preferred by men and women farmers. These findings are consistent with observations of Ashby et al., 1994, Doss and Morris, 2001 who indicate that men and women farmers have different as well as similar preferences related to technologies. It appears that the inherent properties of the hybrids and open-pollinated maize varieties were the drivers of preferences of men or women farmers. Consequently, unravelling the underlying factors influencing varietal preferences by men and women farmers may help develop better breeding strategies that permit the development of gender-response products with greater potential impact on livelihoods in farming households.

The result of the present study does not provide support for a dualistic classification of drought tolerant maize varieties and hybrids as merely “men versus women” preferred products (see Okali, 2012, Glover et al., 2016). The fact that both men and women farmers preferred the same varieties and hybrids in our study provides private seed companies with an opportunity to market the same product to farming communities for both production and home consumption.

## 4 Discussion

Doss and Morris, 2001, Doss, 2002, Ashby et al., 1994 underline the importance of including rural women in the evaluation of varieties, whether the crop is grown for household consumption or for marketing. They emphasise the challenges women farmers face, more than their men counterparts, in terms of unequal access to complementary inputs that diminish their capacity to adopt technologies at the same rate as men. Adequate measures should thus be undertaken by agricultural research and development programmes to address these challenges to ensure equitable impacts of technological change in agriculture (Ashby et al., 1994, Doss and Morris, 2001).

The analyses of trait preference studies highlight the presence of both common and specific characteristics appealing to men and women farmers. It is evident that the preferred characteristics generally fall into production and consumption categories. Drought tolerance, yield and its related characteristics, maturity period, characteristics associate with ease of harvesting as well as source of firewood and feed for domestic animals have been preferred by both men and women farmers. In the meantime, gender-specific differences in characteristics preferences were also apparent with men focusing more on characteristics associated with marketability whereas women focusing on grain colour, taste and appearance.

The result of this study does not provide any clear indication about whether the products developed under the DTMA and STMA projects favoured or disfavoured men or women farmers. The evidence highlights that considerable progress has been made under these projects in generating and delivering drought tolerant maize varieties favoured by both men and women farmers for cultivation. It is therefore fair to acknowledge that the breeding product pipelines are on the right track but need to be adjusted by including additional characteristics preferred by women in collaboration with food science and other disciplines during the development of products. Abate et al. (2017) highlighted the strong desire expressed by farmers to grow hybrid maize with high yield potential and drought tolerance that are endowed with consumption related characteristics. They suggested that maize breeding should consider a diversity of characteristics beyond drought tolerance and grain yield, including an array of production, processing and consumption attributes that are valued by farmers.

This study also underlines the importance of considering specific characteristics that relate to postharvest, nutritional, and processing qualities for food preparation. These have implications for future gender-relevant research in maize breeding and we suggest more research investment to meet these needs and to generate data that will help understand the socially-embedded processes and dynamics of technological change (Glover et al., 2016). The way forward is a prioritized package of traits and postharvest related characteristics that should be included in gender-specific product profiles. An additional component of the strategy should focus on strengthening partnerships with seed companies to encourage them establish an incentive structure for investing in gendered product development. Such a partnership should include assessing the rationale for developing a system of seed certification of products possessing preferred traits and related characteristics as a matter of social responsibility.

Our study underscores the need for strengthening partnerships between breeding, postharvest technology, food science, extension and the private sector. Quality seed is critical for the development of agricultural input markets (Thiele, 1999; Doss and Morris, 2001, Palmer, 2005, Louwaars and de Boef, 2012, McGuire and Sperling, 2016). As indicated by Freeman and Kaguongo, 2003, Awotide et al., 2015, our study advocates for a vibrant and efficient seed sector that can take up maize varieties and hybrids with preferred traits for multiplication and widespread distribution to men and women farmers. The extent to which quality seed of improved varieties can effectively deliver their potential benefits depends on the organization and coordination between key value chain actors, including crop breeders, food science technologists, postharvest specialists, seed companies and producers (Doss and Morris, 2001, McGuire and Sperling, 2016. This further demands an innovative partnership with the private seed sector to ensure seed is readily available to poor and non-poor, men and women farmers at an affordable price.

## 5 Conclusions

The analysis of results has provided justification to support the continued multiplication and commercialization of drought tolerant maize varieties with the top three traits and consumer-based characteristics preferred by both men and by women farmers in the project’s areas.

The analysis indicates that although breeding varieties to match the preferences of target clients is a practical way to facilitate the uptake of new maize varieties, linking specific traits and postharvest processing characteristics with the preferences of women or men farmers is not straightforward. This contradicts what we already know about gender-specific crop and variety preferences because classifying trait-based varietal preferences into those of men or women does not seem to fit with the reality of agricultural practices in farming communities. Such an over-simplification has limited value in influencing breeding and agricultural policy adjustments to address gender concerns, nor will it provide tangible guidance to make any required adjustments to breeding and improved packaging of maize technologies.

One of the lessons drawn from this research is the need to make an adjustment in breeding coupled with effective collaboration with food science and postharvest specialists as well as private seed companies on the packaging of technologies to reach both men and women farmers**.** The gender analysis of sex-disaggregated data captures the top ranked traits preferred by men and women farmers. Such analyses would guide monitoring and evaluation of project objectives as well as assess whether adjustments made in maize breeding pipelines have achieved both availability and equitable dissemination of maize varieties with gender-relevant traits.

Breeders certainly evaluate cost-effectiveness and desirable returns on investment to justify initiation of new and differentiated product development. The size of market segments and number of resource-poor farmers, including women in each segment together with the associated potential for growth and profitability and returns on investment matter for both breeders and private seed companies. Seed companies need to be engaged and encouraged to invest in target group studies and to use socio-economic and gender indicators as a basis for segmentation of their markets. Such a shift in approach may encourage multiple-site focused market studies as a basis for information sharing and the effective linking of breeding programmes with product development and marketing strategies of seed companies.

The gender concern arises when attention is not paid to women’s unique trait preferences during product development. Attention to these preferences is required to improve the likelihood of adoption of maize varieties by women farmers. This study indicates that gender is but one of the required parameters to measure social differences related to packaging of preferred traits in maize varieties and hybrids. Other significant parameters such as ecological factors, socio-economic variables and policy-related matters need to be addressed as integral components of gender analyses. Separate breeding pipelines cannot be developed based upon gender alone Gender related characteristics should be weighted and combined with other diverse user preferences. Such insights can effectively help package gender preferred traits or characteristics and other consumer-based characteristics into maize varieties and indicate how these can be combined with other necessary factors to improve food security and poverty reduction (see Abate et al., 2017).

More knowledge about changes in priorities of both men and women farmer’s coupled with the monetary and other costs (Smith et al., 2017; Orr et al., 2017) may require investments in market segmentation involving agro-ecology, geographic location of markets, as well as other demographic and behavioural factors. Such analyses would inform the attractiveness of the market in terms of measurable customers, profitability, response to differentiated products and stable returns to justify investment.

Finally, the study concludes with a recommendation on the adjustment of maize breeding product pipelines and on forging partnerships and collaboration with extension and the private seed sector. Breeders need to be guided by studies that identify market segments with clearly defined consumer characteristics and unique sets of preferred traits of men and women farmers as well as those of consumers (Orr et al., 2017). Such knowledge will help breeders match their product profiles with the needs of their customers.

## Declaration of Competing Interest

The authors declare that they have no known competing financial interests or personal relationships that could have appeared to influence the work reported in this paper.
